# Unified Theory of Bacterial Sialometabolism: How and Why Bacteria Metabolize Host Sialic Acids

**DOI:** 10.1155/2013/816713

**Published:** 2013-01-15

**Authors:** Eric R. Vimr

**Affiliations:** Laboratory of Sialobiology, Department of Pathobiology, University of Illinois at Urbana-Champaign, Urbana, IL 61802, USA

## Abstract

Sialic acids are structurally diverse nine-carbon ketosugars found mostly in humans and other animals as the terminal units on carbohydrate chains linked to proteins or lipids. The sialic acids function in cell-cell and cell-molecule interactions necessary for organismic development and homeostasis. They not only pose a barrier to microorganisms inhabiting or invading an animal mucosal surface, but also present a source of potential carbon, nitrogen, and cell wall metabolites necessary for bacterial colonization, persistence, growth, and, occasionally, disease. The explosion of microbial genomic sequencing projects reveals remarkable diversity in bacterial sialic acid metabolic potential. How bacteria exploit host sialic acids includes a surprisingly complex array of metabolic and regulatory capabilities that is just now entering a mature research stage. This paper attempts to describe the variety of bacterial sialometabolic systems by focusing on recent advances at the molecular and host-microbe-interaction levels. The hope is that this focus will provide a framework for further research that holds promise for better understanding of the metabolic interplay between bacterial growth and the host environment. An ability to modify or block this interplay has already yielded important new insights into potentially new therapeutic approaches for modifying or blocking bacterial colonization or infection.

## 1. Introduction

At least at some level common experience indicates to almost everyone that life is constrained by competition for limited resources. Formally trained biologists understand this competition as central to evolution, the only fundamental theory in biology. For some microorganisms competitive success in colonizing a mammalian or avian host depends upon specialized metabolism that may support growth in only certain niches. For example, Freter [1] has summarized his own and the work of others by describing the mechanisms of association of bacteria with mucosal surfaces. These mechanisms include “(a) chemotactic attraction of motile bacteria to the surface of the mucus [layer], (b) penetration and trapping within the mucus [layer], (c) adhesion to receptors…, (d) adhesion to epithelial cell surfaces, and (e) multiplication of the mucosa-associated bacteria.” The combined set of traits or phenotypes expressed by a given bacterium defines its potential “virulence factors” or relative colonization success [1, 2]. In the current paper the final stage of the host-microbial interaction is exclusively focused upon multiplication of bacteria at mucosal surfaces. This focus further emphasizes *Escherichia coli *as the predominant facultative anaerobe in animal hosts and its metabolic uses of host sialic acids for nutrition or surface decoration. The narrative approach is intended to support a unified set of observations and hypotheses that could guide future research in the field designated microbial sialobiology [3].

**Figure 1:**
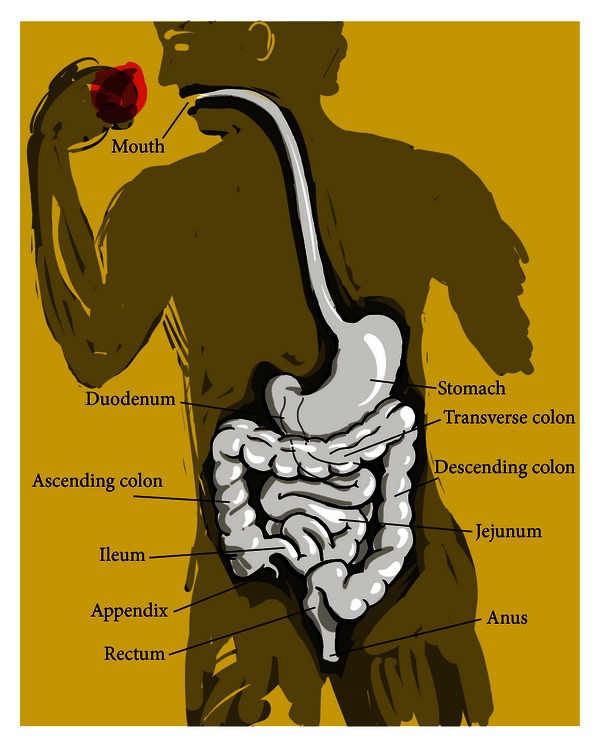
Human gastrointestinal tract. Note that the large intestine (colon with appendix) is permanently colonized by enteric bacteria, *Streptococcus faecalis*, bacteroides, bifidobacteria, eubacteria, peptococci, peptostreptococci, ruminococci, clostridia, and lactobacilli.

By understanding the metabolic use of a single group of prevalent mucosal sugars, the sialic acids, it may be possible to at least partially identify factors controlling which bacteria colonize only certain areas of the gastrointestinal tract or other mucosal surfaces. This goal is central to understanding microbial colonization in disease and health of humans and livestock [2]. The gastrointestinal tract (GIT) is essentially an open tube containing a few valves located between mouth and anus and open to the environment at either end (Figure 1). Bacterial colonization begins during and after birth and may continue to change or become restructured over time as influenced by complicated factors such as diet, overall health, and even geographic location. Other mucosal or epithelial surfaces include the eyes and some sites not shown in Figure 1 like the nasopharynx, lungs, bladder, vagina, and urethra. Some of these sites are normally sterile, for example, eyes, lungs, and bladder unless colonized during an ongoing infectious disease process. Each of these extra-GIT sites expresses a variety of sialic acids that probably do not differ greatly from those found in the large intestine, though much less work has been done on this topic than on the mucus layer and epithelium of the animal large intestine. Regardless of the relative disparity in detailed information between datasets, information about the large intestine should facilitate generalizations to all mucosal sites in healthy and diseased states where microbial involvement is known or suspected. Note that listed in the legend to Figure 1 are bacteria that permanently colonize the large intestine. This group must be in constant competition thus separating the colon from normally sterile sites that usually remain uninfected or, when infected, it is usually by a single species resulting in either clearance by or death of the host. 

Most information about the pathogenic and commensal gut bacteria comes from standard (sometimes referred to as classical) methods of microbial culture and measurements of nutrient use. More current methods such as high-throughput sequencing for identifying both cultivable and noncultivable bacteria as well as nuclear magnetic resonance spectroscopy or mass spectrometry for identifying hundreds of small molecules in complex samples are generating datasets for statistical analyses [4]. However, when the exact identities or functions of important nutrients are unknown, or the metabolic pathways needed for their metabolism are not described, it is unclear how the more recent methods will offer many new insights until supported or refuted by direct experiments after the necessary basic pathways have been elucidated. Therefore, and at the risk of being repetitive, the goal of this current paper is to understand the metabolism of a remarkably distinct, chemically varied, and prevalent family of mucosal sugars that are known in some cases and hypothesized in others to influence in minor-to-major ways the capacity for bacterial niche specialization or disease potential. Some of the ways this information could be applied to specific practical (therapeutic) uses have been described [5].

## 2. Stage Dynamics and Dramatis Personae: GIT Morphology and Bacterial Inhabitants

**Figure 2:**
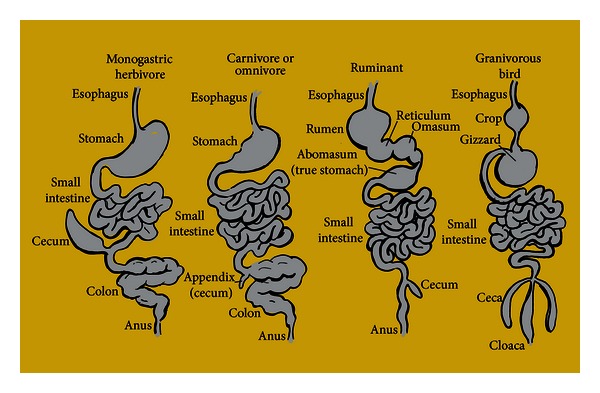
Intestinal structures reflecting different animal dietary types. Examples of monogastric herbivores are horse, rabbit, rat, and pig; carnivore or omnivores are cats, dogs, and man; ruminant examples are cow and sheep while granivorous bird examples are chickens or turkeys. The diagram is modified from reference [6].

Anatomic variation between GITs reflects the digestive needs of a given animal species. Carnivores such as cats and dogs, or human omnivores have a less developed cecum (the appendix) than monogastric herbivores, ruminants, or granivorous birds (Figure 2). Despite this and other anatomic differences most of the digestion and absorption of foodstuffs occurs in the animal small intestine such that carbohydrate, protein, and fat are all digested and mainly absorbed from this site before the undigested residuum empties into the colon [6]. The many factors limiting or selecting for bacterial diversity in most anatomical compartments is in stark contrast to the large intestinal microbiota in its richness and depth of both permanent and occasional inhabitants (Figure 1). 

Table 1 lists some of the bacterial species isolated from the healthy human intestine [6]. The genus/species designations given to some bacteria have changed over time, and other species unique to nonhumans (Table 2) expands the diversity of bacteria residing within animal GITs. Despite the enormous numbers of intestinal bacteria estimated at 10^14^ [7], and the wide species diversity of the colonic microbiota, two facts emerged from standard analyses of the major cultivable bacterial groups [6]. First, *E. coli* is 10 to 100 times more prevalent than clostridia, streptococci, or lactobacilli and one million times more common than yeasts, while 50 to 1000 times less prevalent than bacteroides in the normal human cecum or feces. Second, *E. coli* is found in the rumen and abomasum of cows and crop of chickens as well as the stomachs and entire small intestines of pigs, chickens, cats, and humans living in tropical environments [6]. A discussion of factors limiting *E. coli* to the terminal ileum and colon of healthy humans from temperate climates will not be attempted. Neither will an attempt be made to either support or refute metagenomic analyses that suggest many more, uncultivable bacterial species may exist than those species already identified by standard procedures (Tables 1 and 2). However, and for the purposes of this paper, it is essential to note the guiding principle suggested by the above data. Namely, bacteroides outcompetes *E. coli* by nutritionally exploiting residual foodstuffs not already absorbed by the host including carbohydrates that are undigestible by *E. coli*, while *E. coli* outcompetes all other enterics or other major bacterial groups by mechanism(s) unknown. This paper will address a hypothesis that could explain the evolutionary success of *E. coli*. Failing that goal, the present paper will at least provide a coherent assessment of recent data explaining how bacteria metabolize a major group of host-derived metabolites.

If the central hypothesis introduced above is correct, which nutrient(s) does *E. coli* exploit for survival and outcompeting most of its rivals? Therefore, the conceit of this paper is that *E. coli *has evolved to efficiently exploit host-derived nutrients, and its success as the preeminent facultative large intestinal anaerobe is at least partly owed to an evolutionarily optimized use of host-derived sialic acids. Anyone interested in exploring the genesis of this paper's central hypothesis should read the delightful treatise by Koch [9]. In this work Koch argues, on the basis of biophysical data, at least a partial explanation for how *E. coli* became evolutionarily successful. Indeed, it is in this author's opinion that Koch's article is the single best of all possible articles on the subject of evolutionary success by suggesting that *E. coli* could be the most highly evolved species on the planet.

## 3. Sialic Acids and Their Distributions in the Large Intestine

The sialic acids is a designation given to a group of over 40 naturally occurring nine-carbon keto acids found mainly in animals of the deuterostome embryonic lineage (starfish to humans). These sugars are synthesized rarely by bacteria, and then mostly by pathogens that use sialic acids to masquerade as immunological self, not at all in plants or protostomes except for perhaps a few larval insect stages, and probably not by fungi though the jury remains out in this case [3]. Therefore, when speaking of sialic acid metabolism (sialometabolism) the process is limited to mostly bacterial species that exist as animal commensals or pathogens [3, 10]. Faillard [11] covered the early history of sialic acids since their discovery in the 1930s to the modern era beginning around 1985. Chemists conducted most research during the initial stage of sialic acid discovery. However, Roland Schauer was an early proponent during this time of a different or at least more expansive view of sialic acids, as he clearly recognized that their unique chemical structures and skewed phylogenetic distribution was likely to be significant to diverse biological phenomena. His many insights helped lead to the modern field of sialobiology as a subset of glycobiology and ultimately to the current view of microbial sialobiology described in this paper. Indeed, Schauer was the first to show that a bacterium, *Clostridium perfringens*, appeared to have some mechanism for metabolizing sialic acid in the bacterial growth medium [12]. 

**Figure 3:**
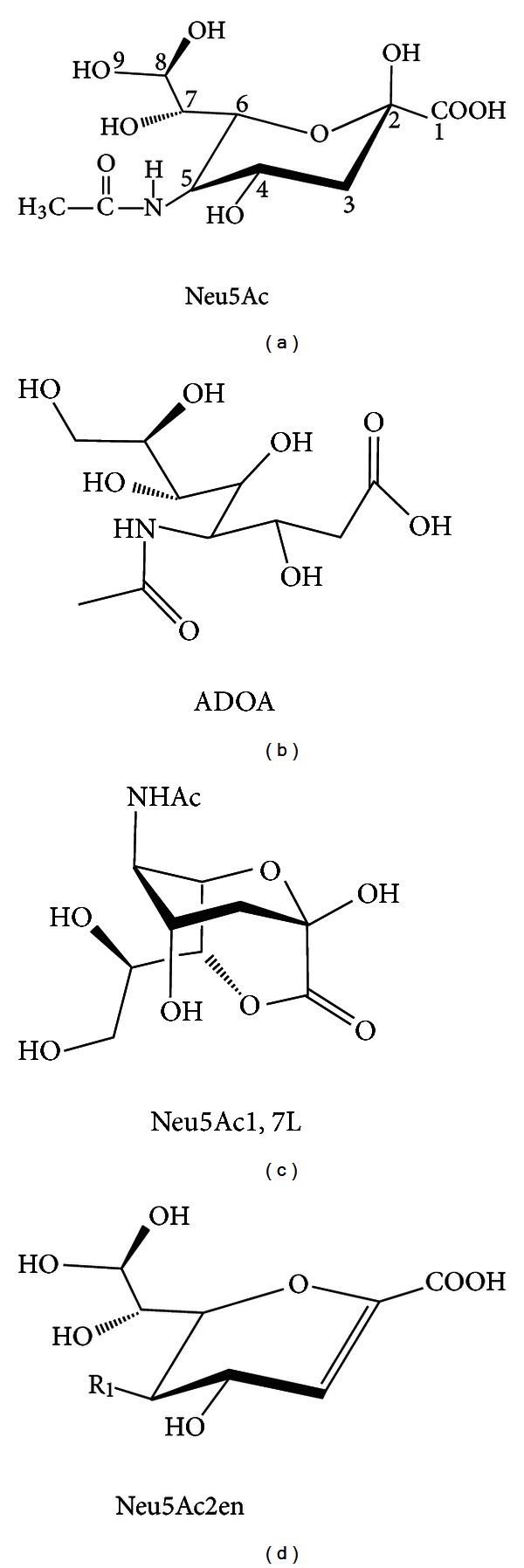
Sialic acid structural diversity. (a) Neu5Ac, the most common sialic acid. The 4 and 7–9 hydroxyls may all be substituted with acetyl groups or less commonly methy, lactyl, succinyl, or phosphate groups. A hydroxyl group on the C-5 acetamido yields Neu5Gc, which is common in all higher animals but humans. (b) Oxidized sialic acid. (c) Lactone detected in high amounts in humans. (d) Anhydro sialic acid and transition state analog of sialidases. Neu5Ac and its derivatives and Neu5Ac1,7L exist bound to other sugars on oligosaccharides of mucin and other glycoconjugates, while ADOA and Neu5Ac2en are free in solution and thus missed by most structural analyses.

The most common sialic acid, 2-keto-3-deoxy-5-acetamido-D-*glycero*-D-*galacto*-nonulosonic acid, is abbreviated Neu5Ac reflecting the backbone neuraminic acid ring, the acetamido group at the carbon position 5, and the glycerol tail composed of carbons 7–9 (Figure 3(a)). Various chemical groups attached to the glycerol tail or ring carbon hydroxyl groups define most Neu5Ac derivatives. By far the most common derivatives bear O-acetyl groups at carbon positions 4, 7, 8, or 9. These additions are catalyzed by O-acetyl transferases in both bacteria and eukaryotes using acetyl-coenzyme A as acetyl donor. O-acetylated sialic acids are abbreviated Neu4(7,8,9),5Ac_2  or  3_ to reflect the position(s) of the acetyl ester(s). *N*-glycolylneuraminic acid (Neu5Gc), synthesized by a hydroxylase that adds a hydroxyl group to the carbon-5 acetamido of Neu5Ac (Figure 3(a)) is the other major form of sialic acid in most animals other than humans. Its absence in humans is due to a null mutation in the hydroxylase gene, indicating gene function was lost after the split of the human ancestor from that of the great apes. Ajit Varki and his colleagues have speculated about the biological consequences of Neu5Gc's absence in humans [13], but it is possible that the loss has no major consequence other than one less nutrient for bacteria to exploit in humans. Less common derivatives include an oxidized form of Neu5Ac, 4-(acetylamino)-2,4-dideoxy-D-*glycero*-D-*galacto*-octanoic acid (ADOA), a carbon position 1–7 lactone (Neu5Ac1,7L), and a 2-deoxy anhydro form, Neu5Ac2en (Figures 3(b)–3(d), resp.).

**Figure 4:**
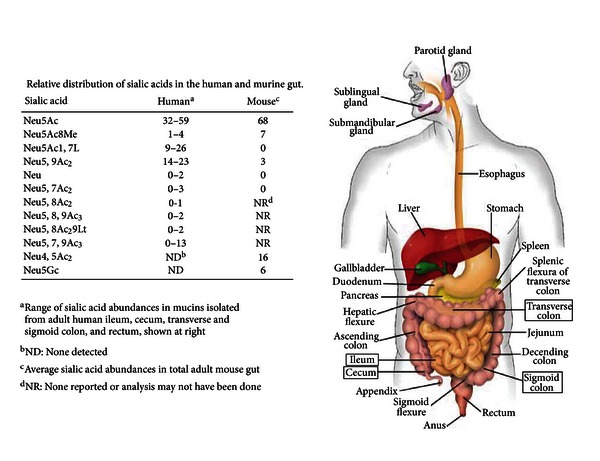
Relative distribution of sialic acids in the human and murine gut. Sialic acid abundances were determined for the human GIT compartments highlighted in rectangles [14]. Mouse values are for the small and large intestine [15].

The structural diversity of the sialic acids is matched by their *regio*-distributional differences along the length of the large intestine [14, 15], revealing an increasing sialic acid gradient from ileum to rectum.  Figure 4 also shows that in humans Neu5Ac, Neu5Ac1,7L, and various O-acetylated forms are the most prevalent sialic acid derivatives. By contrast, the mouse as expected produces Neu5Gc [15], but a possibly lesser amount of the other derivatives found in humans (Figure 4). Except for Neu5Gc it is unclear whether differences between humans and mouse colonic sialic acids reflects true species diversity or artifacts of the sampling and analytical methods used for detection. If these differences were real, the mouse would be a poor model for investigating sialometabolism in humans.

Most sialic acid is linked to other sugars including other sialic acids and the di-, oligo-, or polysaccharides formed by these carbohydrate linkages are attached to lipids (forming glycolipids) or proteins (forming glycoproteins) comprising the group of molecules called glycoconjugates. Sialic acids are frequently, when present in a sugar chain, the terminal sugar linked to subterminal carbohydrate units through glycoketosidic bonds between the carbon-2 hydroxyl of the terminal sugar and subterminal hydroxyls at various positions depending on the acceptor. In the GIT as well as other mucosal surfaces sialic acids are a major component of mucins bathing the epithelial surfaces and the glycoconjugates comprising the epithelial glycocalyx including the glycolipids and glycoproteins bound to epithelial cell surfaces. The types of glycoconjugates and their interactions in health and disease have been recently reviewed [16–18]. Bound sialic acids by definition are unavailable to bacteria unless first released by sialidases (neuraminidase, E.C.3.2.1.18), which hydrolyze the linkages between terminal sialic acids and subterminal sugars. These hydrolases are produced by the host (endogenously) and by some bacterial species (exogenously). Bacterial sialidases come in a great variety of structures and may be multifunctional [19]. The combined actions of endogenous and exogenous (bacterially derived) sialidases is thought to be necessary for any further microbial utilization of host sialic acids for either synthetic or catabolic purposes [3]. The reader is directed to the original literature and reviews describing the molecular characterization of the bacterial sialidase superfamily [19–24], and a more recent review listing further examples of bacterial sialidases [25] for additional background information. 

## 4. Canonical Bacterial Pathway for Catabolism (Dissimilation) of Host-Derived Neu5Ac

**Figure 5:**
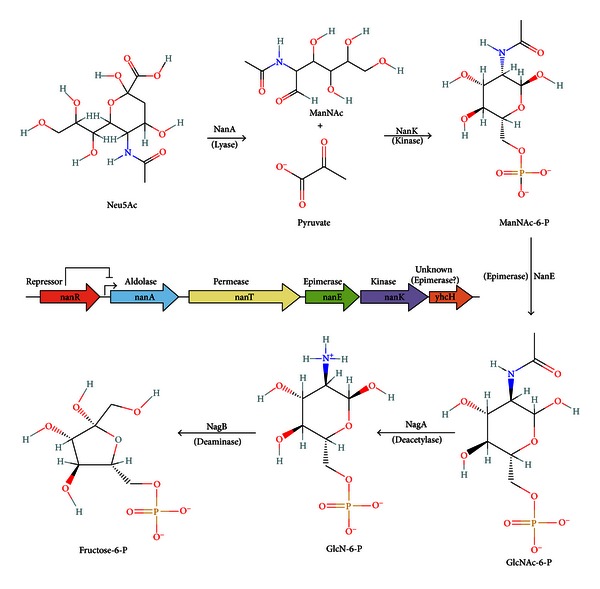
Canonical *N*-acylneuraminate (*nan*) dissimilatory pathway for metabolism of Neu5Ac by *E. coli*. Colored arrows indicate relative transcriptional directions and functions of genes involved in converting Neu5Ac to GlcNAc-6-P after transport of exogenous sialic acid by the permease, NanT (yellow): aldolase or lyase (blue), epimerase (green), kinase (purple), *yhcH* (orange). Expression of the structural genes of this operon are regulated by the repressor, NanR (red) located immediately upstream of the *nanA* start site. Depending on the bacterial species, NagA or NagB may be part of the canonical operon or, as in the case of *E. coli*, located in a separate operon.

Once sialic acids are released by hydrolysis they are available like most other sugars free in solution for transport into the cell and catabolic fermentation or oxidation. That the Neu5Ac catabolic pathway went undiscovered or, indeed, not even thought of until 1985 no doubt reflects the phylogenetic rarity of sialic acid and its commercial expense at the time as an available potential nutrient for experimental testing [26–29].  Figure 5 shows the canonical *E. coli* sialocatabolic operon (in color) and upstream repressor gene *nanR*. Induced catabolic genes, their encoded polypeptides, and functions where known are also depicted in the figure. Once transported into the cell by NanT the lyase encoded by *nanA* releases *N*-acetylmannosamine (ManNAc) and pyruvate; the latter enters the oxidative TCA pathway of energy production. NanK phosphorylates the ring form of ManNAc yielding the 6-phosphate derivative ManNAc-6-P. NanE converts ManNAc-6-P to *N*-acetylglucosamine-6-phosphate (GlcNAc-6-P), the inducer of the *nag* operon, with deacetylation and deamination by NagA and NagB, respectively, completing Neu5Ac dissimilation to fructose-6-phosphate. Neu5Ac thus serves as a carbon and nitrogen source, a source of energy, and a source of precursors for cell wall synthesis. With minor variation [30], the canonical biochemical pathway exists in all microorganisms known to catabolize Neu5Ac. Variations in gene organization are widespread in different species, but utilization of Neu5Ac for growth depends on some form of NanATEK and NagAB. The function of YhcH is unknown, but as discussed below it is hypothesized to function in dissimilation of sialic acids other than Neu5Ac, since deleting *yhcH* had no effect on growth of *E. coli* on Neu5Ac [31].

## 5. The Good, the Bad, and the Ugly-Evidence for or Against the Involvement of Sialometabolism in Bacterial Colonization and Pathogenesis

Bacteria such as *E. coli* serotype K1 and some neisserial serotypes synthesize sialic acids de novo and assemble them into cell surface glycolipids [32]. In some cases, the capsular polysaccharides synthesized by these bacteria mimic host molecules and thus fail to elicit a host immunological response [3]. In other sialo-positive serotypes the polysaccharides have no host analogs due to linkage differences and so form the basis of effective vaccines against bacterial meningitis. In all animal models tested loss of capsular sialic acids results in attenuation, indicating the essential role of the capsule in pathogenesis. The ineluctable conclusion is that bacteria presenting sialic acids at their surfaces do so as a mechanism of avoiding host immune surveillance or to otherwise mask the bacterial surface making it less susceptible to host defense mechanisms both innate and acquired. However, antibodies to capsules if present as a result of prior infection, passive transfer, or vaccination protect against disease, at least in the short term.

Another group of bacteria displaying surface sialic acid though lacking the de novo metabolic pathway include *Neisseria gonorrhoeae*, *Haemophilus influenzae* and other *Haemophilus* spp., and *Pasteurella multocida*. These bacteria acquire sialic acids from the host using either surface sialytransferase or hybrid synthetic-catabolic pathways including sialic acid transporters and a truncated synthetic system using only the activating enzyme and a sialyltransferase [33]. Experimental evidence in a natural *P. multocida* host, the cow, and a mouse model of invasive disease indicated that sialic acid transport was essential to pathogenesis [34]. Using substantially the same approaches, sialic acid uptake was independently confirmed to be essential in turkey pathogenesis, another natural host of this facultative pathogen [35]. A deletion of *nanA* did not affect *P. multocida* pathogenicity [34], suggesting that catabolism of host sialic acids was not essential to virulence. Similarly, *nanA* was unessential for colonization of the mouse nasopharynx by *H. influenzae* [36]. The combined results described above unambiguously support an essential function of bacterial sialic acid decoration for evading immune responses but provided little indication that an ability to catabolize these sugars was important to either colonization or disease. However, as discussed below, studies in other bacteria strongly suggest that host sialic acid catabolism has at least a minor role in pathogenesis in different species, and possibly a major role in colonization. None of the bacteria to be discussed below either synthesize sialic acid de novo or use a truncated catabolic-synthetic pathway for surface decoration. Therefore, the sole function of sialocatabolism in these bacteria must be growth at the expense of host mucosal sialic acids.

A variety of studies have suggested sialocatabolism is important to colonization or fitness in animal hosts. However, none of the studies has been independently confirmed, and some of the effects of deleting *nanA* or the sialate uptake system have shown less than dramatic effects on fitness. For example, deleting the *nanA* orthologs in *Vibrio cholerae* suggested a minor (<20-fold) decrease in competitive index when compared to wild type [37]. Single-infection experiments showed no significant difference with wild type, except at one early sampling interval [37]. A greater fitness effect (50–100 times less) was reported for a *nanA* deletion in *Vibrio vulnificus*, and a 500-fold increased LD_50_ after intraperitoneal injection in mice [38]. However, neither of the above studies rule out whether the effects were caused by an inability to metabolize sialic acids or toxicity resulting from intracellular sialic acid accumulation in the *nanA* mutants [26]. Jeong et al. [38] indicated there was no toxicity observed in vitro, but this statement was contradicted experimentally in a later study by some of the same authors [39], making the in vivo results suspect or at least requiring independent confirmation before they can be taken seriously. Furthermore, a *nanA* mutation in an uropathogenic strain of *E. coli* had no fitness defect in mouse bladder or kidneys, consistent with the effect of peptide or amino acid utilization in these extraintestinal sites [40]. However, some of the same authors later showed a 50-fold decreased fitness of an uropathogenic *E. coli nanA* mutant during bacteremia [41]. Again it is unclear whether this effect is due to sialic acid toxicity or lack of its contribution to growth under in vivo conditions. Much more work is needed before any conclusions can be drawn from these studies that do, however, at least point to either a nutritional or detoxifying effect of sialate aldolase (NanA) in bacterial-host interactions.

By constructing a double mutant defective in sialate uptake and aldolase, one can experimentally control for both the nutritional and antitoxicity functions of bacterial sialocatabolism. Using an *E. coli nanAT* double mutant in streptomycin-treated mice the mutant was 500–1000 times less able than wild type to colonize the host, consistent with a previously reported potential role of sialocatabolism in mucin utilization [42, 43]. Interestingly, enteropathogenic *E. coli* O157 did not appear to use sialic acid for colonization, which was one of the sugars used by commensal *E. coli*, suggesting sugars not used by the resident population support colonization of the pathogenic strain. In a recent study *E. coli* passage through the mouse intestine selected for derivatives with increased metabolic efficiency, including genes controlled by the NanR sialorepressor [44]. The problem with these otherwise elaborate studies [41–44] is that the mice are treated with streptomycin to reduce the normal intestinal microbiota to allow a better chance of introduced strains to colonize. In terms of sialic acid utilization, this treatment means that all or most of the free sialic acid for growth must come from endogenous (host) sialidases, and any residual sialidase-positive bacteria remaining after the drug treatment. This conclusion follows from repeated observations that *E. coli* lacks sialidase and must rely on other providers for free sialic acids in vivo. Uncompromised studies are needed before any firm conclusions can be drawn about the role of sialocatabolism in *E. coli* gut colonization.

A seemingly more convincing study suggesting the role of sialocatabolism in *Streptococcus agalactiae* (group B streptococci, GBS) was recently published [45]. GBS are a leading cause of neonatal meningitis in human newborns and a common inhabitant of the vagina mucosal surface. Except for glucose there are few obvious carbohydrates that GBS can use for energy production. Because GBS lacks sialidase, any source of free sialic acid must come from endogenous or other microbial sialidase activities in the vagina. The authors showed that exogenous addition of sialic acid in a mouse model increased wild type growth in the vagina and had, as expected, no effect on a sialate transport-defective mutant [45]. These results add to the overall hypothesis of the current and earlier paper [3] by suggesting that host-derived sialic acids are important to colonization and disease potential. 

**Figure 6:**
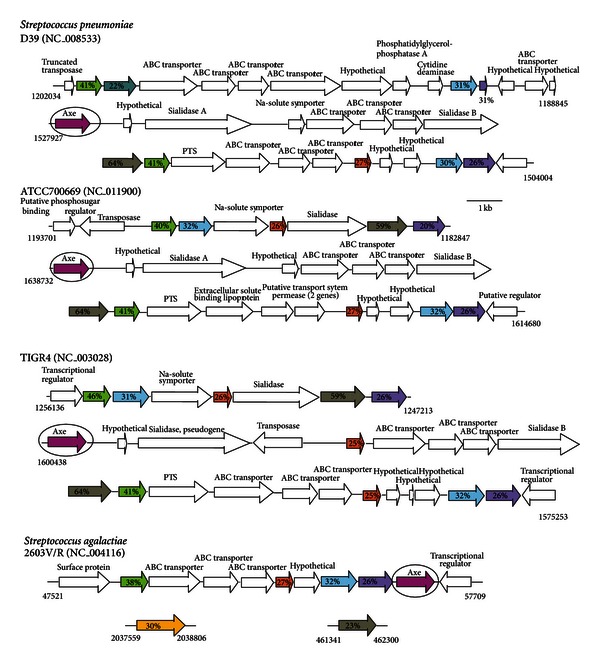
Proposed organization of *nan* gene clusters in selected streptococci. Canonical nan genes in *S. pneumoniae* strains D39, ATCC700669 and TIGR4, and GBS *S. agalactiae* have the same color designations as given in the legend to Figure 5, with the addition of Axe (magenta), YjhC (grey), and YjhB (gold) based on orthologs of *E. coli* genes described in the text. The known or proposed functions of other genes in the various clusters are listed above the open arrows. The numbers below at left or right of the first or last gene in the cluster gives the beginning and ending nucleotide positions of each gene segment.

A study similar to that described above for GBS [45] was previously carried out in *S. pneumoniae* [46]. By contrast to GBS, inactivating the *S. pneumoniae* sialate uptake system had only a 50-fold decreased fitness. However, the dramatic in vivo effects seen with GBS were only observed when exogenous sialic acid was injected into the animal model, which is problematic unless the results are compared to the expected general increase in all coresident species utilizing sialic acids in the nares, lungs, or vagina. In other words, the sialouptake defect in GBS had little or no effect on colonization in any of these sites relative to wild type unless exogenous sialic acid were coadministered, which is the expected result essentially making the mouse an unnecessary “furry test tube.” Both streptococcal studies [45, 46] also can be criticized on the basis of genomic comparisons of sialocatabolic loci in *S. pneumoniae*.  Figure 6 shows the known or predicted sialocatabolic genes in three sequenced strains: D39, one of the original Avery isolates, ATCC700669, and TIGR4. Despite a few differences in overall gene arrangement the gene duplications or triplication of *nan* orthologs *nanA* (lyase, blue), *nanE* (epimerase, green), *yhcH* (unknown, orange), *yjhC* (unknown, grey), and *nanK* (kinase, purple) point to past recombination events in the streptococcal sialocatabolism regions of these strains (Figure 6). Of note from this analysis is the *nanA* orthologs of strain D39 bear identical point mutations early in the sequence resulting in an inability to catabolize exogenous sialic acids. Despite this defect D39 is as pathogenic for mice as TIGR4 or other wild type streptococcal strains indicating that a natural sialocatabolic-defective mutant might be unaffected for colonization or disease potential. Work is in progress in my laboratory to resolve the contradictory evidence, which includes one other study claiming *S. pneumoniae* D39 uses sialic acid derived from hog gastric mucin for growth [47]. 

As discussed above, *H. influenzae* and *P. multocida* catabolize sialic acids and sialic acid transport is essential for virulence while use of sialic acid as energy source is not [33–36]. These findings were confirmed and extended in vivo for nontypeable *H. influenzae* (NTHi), an important agent of middle ear (otitis media) infections especially in children [48, 49]. Thus, unlike *E. coli* and possibly GBS and *S. pneumoniae*, an ability to catabolize host-derived sialic acids might not necessarily correlate with colonization or pathogenesis. The regulatory mechanism controlling *H. influenzae* sialic acid uptake and catabolism is similar to that described previously for *E. coli* [31, 50–52]. However, the importance ascribed to this regulatory system [50–52] has been recently challenged [49]. Regardless of the discrepancies, another area where host-derived sialic acid may be important to NTHi is biofilm formation under both in vivo and in vitro conditions [53–56]. While these studies support a role for sialic acid in biofilm formation in vitro, the entire concept of NTHi biofilms in the middle ear and by extension the role of host-derived sialic acids in otitis media has been challenged [57]. The discrepancies between groups investigating substantially identical phenomena using similar methodologies warrants caution when extrapolating in vitro results to in vivo conditions. Even in vivo results may be misleading when the relevance of the animal model might be flawed.

Other bacteria where biofilms and sialic acids might be important to infection include *Pseudomonas aeruginosa*, an environmental opportunist, and *S. pneumoniae*, an important cause of ear infections, meningitis, septicemia, and pneumonia in especially young, old or immunocompromised human beings. Both microorganisms express sialidase(s) at their surfaces, although the *P. aeruginosa* enzyme seems to cleave sialic acid-like molecules (pseudaminic acids) found on a variety of bacterial species including *P. aeruginosa *[58], but not animals of the deuterostome lineage [59]. Furthermore, and unlike *S. penumoniae*, *P. aeruginosa* lacks the catabolic genes to transport or metabolize sialic acids. However, in both bacterial species sialidase seems to be required for biofilm formation in vivo [60–62]. Competitive sialidase inhibitors that bind to the respective enzyme's active sites appeared to reduce biofilm formation and in vivo fitness, suggesting that these inhibitors, normally prescribed for viral influenza infections, may be useful clinically for treating pseudomonad and streptococcal infections. Similar to biofilm formation in NTHi, where host-derived sialic acid presumably influences biofilm formation by incorporation into bacterial surface structures, the pseudomonad sialidase might modulate pseudaminic acid levels on bacterial surface structures thereby promoting or inhibiting biofilm formation. The streptococcal situation is much more complicated, not least by conflicting evidence showing an effect of Neu5Ac but not Neu5Gc on biofilm formation when contaminating amounts of Neu5Ac in the Neu5Gc used was probably in excess of the effective Neu5Ac concentration [62]. Furthermore, *S. pneumoniae* expresses up to three sialidases each producing a different hydrolytic product [63]. More work is obviously needed to confirm the potentially exciting findings, especially when competitive sialidase inhibitors might form the basis of a useful therapeutic approach. For example, the major sialidase expressed by all strains of *S. pneumoniae* has been linked to phase-variation during infection and modification of the leukocyte inflammatory response [64–66], supporting the possibility of a general approach aimed at blocking sialidase activity.

## 6. Mechanisms of Bacterial Acquisition or Scavenging of Host Sialic Acids

As indicated throughout the current paper sialic acids are present in free form at low amounts presumably resulting from the actions of endogenous sialidases. At least four forms of human sialidase have been identified with one located at the plasma membrane [67]. In principle any one of the endogenous sialidases could gain access to mucosal sialoglycoconjugates and release free sialic acid product. In complex microbial communities like those at mucosal surfaces, bacteria express a wide variety of sialidases that can either be excreted, surface-associated, intracellular, or periplasmically located. For example, Mizan et al. [68] showed that *P. multocida* uses its two different surface sialidases to grow on different sialoglycoconjugates by releasing free sialic acid for transport and catabolism by products of the sialocatabolic operon [34]. However, the complexity of sialometabolism at mucosal surfaces is likely to be greater than a simple scavenging model might otherwise indicate. 

**Figure 7:**
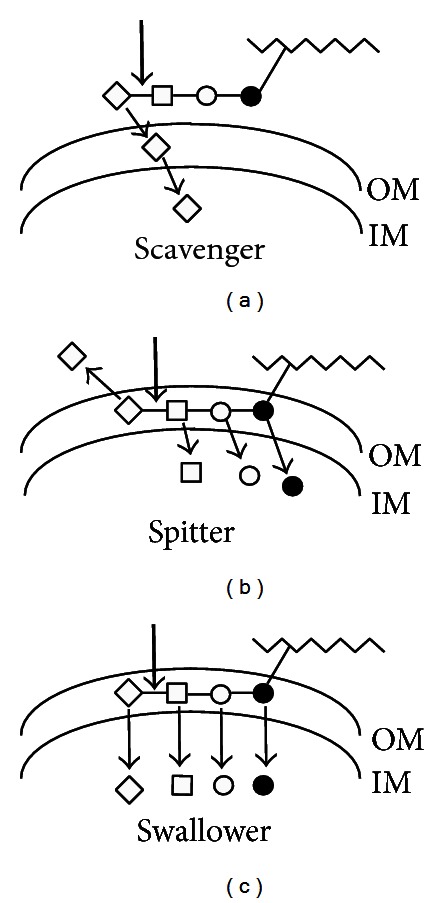
Modes of acquisition of host sialic acids by typical Gram-negative bacterial species. (a) Bacteria with a scavenger mode of sialic acid acquisition depend on either their own or another source of sialidase (bold arrows) to release free sialic acid (diamond) from carbohydrate chains linked to host substrates (jagged lines). Released sialic acids diffuse into the periplasm between the outer and inner membranes (OM and IM, resp.,) for transport by specific permease(s) into the cytoplasm. (b) The spitter mode of acquisition involves sialidase release but inability to further metabolize sialic acid. These bacteria then sequentially release GlcNAc (open squares), galactose (Gal, open circles), and *N*-acetylgalactosamine (GalNAc, bold circles) from the idealized oligosaccharide for subsequent dissimilatory pathways. Note that the entire oligosaccharide chain may be degraded within the periplasm. (c) The swallower mode is identical to that of the spitter, except that swallowers catabolize the released sialic acid(s). Note that the scavenger and spitter modes are available to Gram-positive bacteria that lack a periplasmic space.

Consider in addition to simple scavenging of free sialic acids (Figure 7(a)) two models with distinctly different outcomes but both involving sialidases unique to the bacterial species.  Figure 7(b) shows an example of a “spitter” in which terminal sialic acid, as part of a glycoconjugate, is cleaved by a periplasmic sialidase but by a strain otherwise lacking all other sialocatabolic functions [69]. The outcome is a sort of acid reflux whereby the released sialic acid enters the extracellular milieu while the subterminal sugars are subsequently hydrolyzed by specific glycosidases, then transported and used for cell growth. Clearly, this is a growth strategy that sacrifices the sialic acid in turn to gain access to subterminal sugars on carbohydrate chains, underscoring the previous conclusion that the diversity of bacterial sialocatabolic pathways evolved in response to the apotheosis if not emergence of sialic acids in the deuterostome lineage [3]. Therefore, the only difference between a spitter and a “swallower” (Figure 3(c)) is that the latter has a sialocatabolic system to exploit the full richness of carbohydrate substrates. By definition, swallowers like *Bacteroides* spp [30, 70] should not be in competition with *E. coli* unless their sialo uptake systems are so inefficient that they allow sialic acid reflux and consequent scavenging by *E. coli* or other sialidase-negative species. Koch [9] explained how *E. coli* carbohydrate transport systems are evolutionarily optimized for scavenging sugars.

## 7. The Salad Bar-Substrate Availability at Mucosal Surfaces

That some gut bacteria utilize host glycoconjugates for nutrition is strongly supported by an ingenious study looking at the microbiological consequences of feeding neonatal pigs a normal diet parenterally or intravenously (i.v.) bypassing the GIT. Sixty-two percent of the i.v.-fed ileal microbiota were mucolytic species compared to 33% of the species detected from piglets fed parenterally [71]. This result points to the nutritional foraging by GIT bacteria of carbohydrate substrates abundant at the mucosal surface when other food sources are absent. The conclusion derived from this in vivo study is consistent with the extensive carbohydrate-utilization systems in *Bacteroides thetaiotaomicron*, which is dedicated to foraging mucosal surfaces as the bacterium searches for alternative energy sources [70]. The foraging system requires surface-associated glycosidases, outer membrane oligosaccharide transporter, and periplasmic glycosidases to release monosaccharides, inner membrane transporters, and the intracellular metabolic functions to produce energy from the imported sugars. In other words, *B. thetaiotaomicron* is an example of a spitter or a swallower (Figures 7(b) and 7(c), resp.), depending on its ability to metabolize sialic acid. While the above studies identify the nutritional use of enteral carbohydrates for bacterial nutrition, they contribute directly nothing to understanding the metabolism of specific mucosal sialoglycoconjugates. 

Some investigators have demonstrated metabolism of sialylated mucins isolated from various mucosal surfaces. In one study mucins from germ-free rats were incubated with total cecal microbiota from conventionally raised rats. Sialylated mucins were degraded more rapidly than the neutral or sulfated forms suggesting an overall optimized use of sialic acids by intestinal bacteria [72]. Although ocular fluid from many humans is sterile, some studies have shown that other people maintain a commensal bacterial population without incident. These commensals were shown to degrade sialylated ocular mucins indicating the primary carbon and energy sources for these bacteria are carbohydrates found at ocular mucosal sites [73]. Similarly, Burnaugh and colleagues showed that in vitro growth of *S. pneumoniae* on human glycoconjugates relied on the sequential action of several different surface-bound glycosidases, including the major sialidase-A [74]. However, a mutant defective for this sialidase was still able to colonize the mouse lung, suggesting either free sialic acid is not essential to the host-microbe interaction or that other sources of this sugar are to be found in the lung [75]. Alternatively, the contribution of the sialidase to disease might be host species-specific, underscoring the potential pitfall when extrapolating too freely between in vitro and in vivo results.

Probably the best commercially available source of chemically characterized sialomucin for experimental investigation is bovine submaxillary gland mucin (SGM). Sensitive fluorometric methods exist to identify Neu5Ac, all of its O-acetylated derivatives, and Neu5Gc or its derivatives [76]. However, SGM cannot adequately represent the vast variety of carbohydrates detected in human mucins. For example, using electrospray ionization quadrupole time-of-flight mass spectrometry, 46 neutral, and 50 acidic carbohydrate chains were detected from mucin oligosaccharides isolated from the ileum, cecum, transverse colon, sigmoid colon, and rectum [77]. Neutral oligosaccharides do not contain Neu5Ac or sulfate residues while acidic chains included Neu5Ac, sulfate, or both Neu5Ac and sulfate [76]. Anthony Corfield and his colleagues were the first to show that some mucosal bacteria synthesized sialidase, glycosulfatase, and sialate O-acetyl esterase, supporting the idea that acidic sugars are a nutritional source for bacteria residing in the GIT [78]. As these authors noted [78], because sulfated carbohydrates and O-acetylated sialic acids reduce glycosidase activity, bacteria evolved mechanisms to remove the modifications so that the “released” carbohydrates could become more readily available for nutrition. Some in vivo experimental results support this conclusion.

Research with *Capnocytophaga canimorsus*, a member of the *Bacteroidaceae* family, underscores how bacteria feeding off mammalian cell surface glycoconguates gain competitive advantage [69]. However, the authors failed to cite an earlier publication by Michael Malamy and his associates demonstrating essentially the same phenomenon with *Bacteroides fragilis* [79]. These investigators showed that a *B. fragilis *sialidase-negative mutant could not compete against wild type when growing in tissue culture or a rat-pouch model of human abscess. Bacterial growth in both models was equivalent until the time glucose was exhausted, suggesting that the wild type exploited sialoglycoconjugates that were unavailable to the mutant [79]. Both studies [69, 79] focus on the need for increased attention to bacterial sialidase substrate specificities because the variety of sialic acids and their linkages to subterminal sugars is so diverse. For example, using a novel system of chemoselective labeling, Parker et al. [80] showed that the minor *S. pneumoniae *sialidase-C strongly preferred Neu5Ac to Neu5Gc. The paucity of Neu5Gc in humans may in part explain why *S. pneumoniae* is such a successful human pathogen while not generally a problem in other animals. These observations concerning substrate availability further suggest that animal models of human infectious diseases may not accurately report reliable information. The unavoidable conclusion is that testing therapeutics aimed at inhibiting sialometabolism could require human volunteers.

While investigations of sialomucin and other sialoglycoconjugate substrates will continue to expand understanding of bacterial sialometabolism, it seems essential to have a unified theory for at least one bacterium. This theory would include all known and putative sialocatabolic functions thus allowing directed approaches aimed at understanding metabolic pathways while facilitating extrapolation to other sialo-capable bacterial species. *E. coli *remains the best model organism for developing a unified theory of sialometabolism.

## 8. Identification of the *E. coli* Sialoregulon

**Figure 8:**
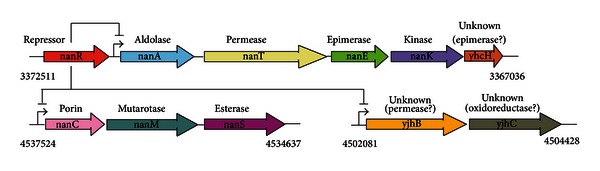
The *E. coli *sialoregulon. In addition to the canonical *nan* operon (with colored arrows having the same designations as given in Figure 6), other genes regulated by NanR include *nanC* (pink), *nanM* (teal), and *nanS* (magenta), which is homologous to the *axe* genes shown for streptococci in Figure 6. Another coregulated operon is composed of a putative permease, YjhB (gold) and oxidoreductase, YjhC (grey).

The *E. coli* sialocatabolic system is regulated by repressor protein, NanR, whose structural gene is located immediately upstream of the *nanATEK*-*yhcH* operon (Figure 8). NanR binds to a unique operator with three GGTATA repeats separated by two or three nucleotides [31]. The *nan* operon responds to exogenous sialic acid with *nanA* induction up to 1000-fold [26], indicating the important function of the lyase for both nutritional use of sialic acids and detoxification [26, 27]. Except for the unknown function of YhcH, the canonical *nan* operon is dedicated to catabolism of Neu5Ac [26–29, 31]. However, when transcriptome analysis of a *nanR* mutant was compared to wild type, or when wild type bacteria were grown with Neu5Ac or glycerol as sole carbon source [3], two additional NanR coregulated operons were identified by their increased message production representing five additional genes (Figure 8). Both *nanCMS* and *yjhBC* include NanR operators upstream of the putative transcriptional start sites for each operon. The functions of three of the five coregulated genes is known or at least supported by some experimental evidence.

The *nanCMS* operon is composed of genes encoding an outer membrane porin (*nanC*), sialate mutarotase (*nanM*), and sialate O-acetyl esterase (*nanS*). The porin is not required for growth of *E. coli* on Neu5Ac unless outer membrane porins OmpF and OmpC are absent [81]. The recently solved crystal structure of NanC confirms its similarity to porins with presumed selectivity for acidic oligonucleotides [82]. The NanC 12-stranded *β*-barrel tertiary structure defines an open pore with average radius of 3.3 Å lined by two strings of basic amino acid residues apposed across the pore. The alignment of basic residues is conserved within a family of diffusion channels that likely facilitates the entry of acidic oligosaccharides [82]. The similarity of NanC to this family of diffusion channels was thought to indicate preferential uptake of sialooligomers [82]. However, there is little indication that such oligomers would exist outside of polysialic acid in the central nervous system [3], nor any known periplasmic or intracellular *E. coli* sialidase that could convert oligomers to free sialic acids. A recent transcriptome analysis of *E. coli *indicated that *nanC* was induced when *E. coli* is growing in biofilms [83]. Given that *nanC* is part of an operon controlled by NanR, it is difficult to see how induction could occur unless the operon was under control of some regulator other than NanR. Interestingly, *nanC* was one of the genes identified by a targeted mutagenesis approach in *Salmonella enterica* serovar Typhimurium strain ATCC14028 as having decreased fitness during competitive mouse infection [84]. The combined results of crystallography, transcriptome, and animal studies strongly suggest that NanC is a porin that is important to host colonization and disease. That it is part of the sialoregulon further suggests it somehow facilitates utilization of host sialoglycoconjugates or at least their released sialic acids.

Like most sugars in solution, the pyranose Neu5Ac ring continuously rotates by opening and closing between the thermodynamically more stable *β*-anomer with axially directed hydroxyl at the carbon-2 position (Figure 1(a)) and the *α*-anomeric form (<10% of the total Neu5Ac in an equilibrium solution) with hydroxyl directed equatorially. The mutarotation time to equilibrium starting from a pure solution of the *α*-anomer is on the order of an hour, such that at equilibrium the mixture contains >90%  *β*-anomer [85]. By contrast to this equilibrium mixture, all Neu5Ac or derivatives attached to glycoconjugates are in *α*-glycoketosidic linkages [86]. Because mammalian and bacterial sialidases are retaining hydrolases, the *α*-isomer is exclusively released from substrates after enzyme cleavage. Since spontaneous rotation is slow, and if as seems logical bacterial sialate transporters recognize the thermodynamically predominant sialate in solution, bacterial mutarotase encoded by *nanM* catalyzing the *α*- to *β*-isomeric sialate conversion may enhance competitive success at mucosal surfaces. Thus, NanM could increase the scavenging potential for sialates in an animal host where bacteria rely at least in part on sugars released by endogenous or exogenous sialidases for growth. This is an attractive idea with some supporting evidence [87]. Mutarotation from *α*- to *β*-Neu5Ac is easy to entertain when the enzyme is located in the periplasm. However, some bacteria have more than one copy of *nanM* suggesting a cytoplasmic location for at least some Neu5Ac mutarotases [87]. A cytoplasmic location for mutarotase is problematic because the lyase encoded by *nanA* requires *α*-Neu5Ac substrate (Figure 3(a)). It is conceivable that NanA pulls the *β*-anomeric form, presumably the form transported by NanT, in the direction of the *α*-isomer. However, at best, there would seem to be competition between cytoplasmic NanM and NanA. Therefore, because Neu5Ac accumulation in the cytoplasm is potentially toxic [26], perhaps NanM functions primarily as a detoxifying enzyme in the event that *α*-Neu5Ac is the toxic form. In any case, NanM and its predicted orthologs are found in many but by no means all bacterial species with known or predicted canonical Neu5Ac dissimilatory pathways (Figure 5), suggesting the mutarotase is not an essential component of sialocatabolism. Indeed, an *E. coli nanM* mutant had at most a 20% reduction in growth rate relative to wild type under experimental conditions favoring overabundance of the *α*-anomeric form [87], as might exist while bacteria scavenge Neu5Ac in their natural hosts (Figure 7(a)). This relatively modest growth defect might be, however, a significant factor helping to explain part of the overall puzzle why or how* E. coli *became the preeminent facultative anaerobe in the GIT. One obvious test would be to construct an *E. coli nanM* mutant and compare its fitness to wild type in an appropriate animal model. Unfortunately, as discussed above, it is not entirely clear what an appropriate model would be unless the phenotypic effect in, say the mouse, were a dramatic one.

**Figure 9:**
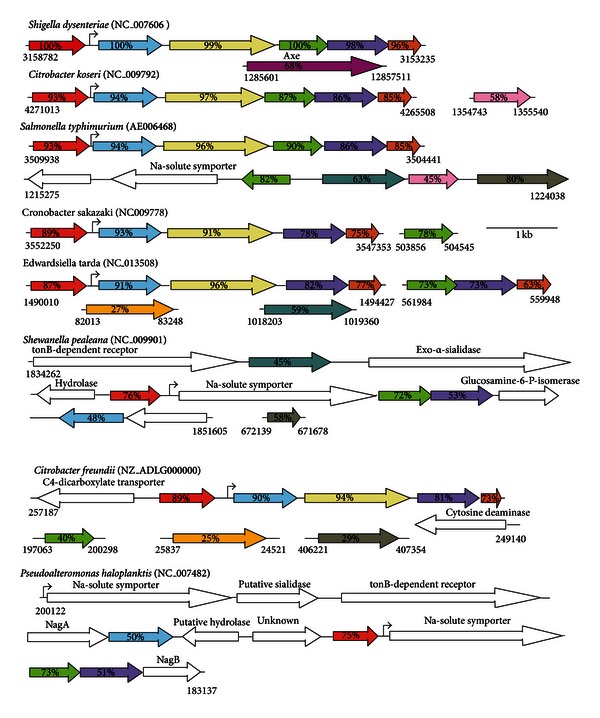
Bacteria with putative *nan* genes coregulated by NanR. Colored and open arrows have their same designations as given in Figures 6 and 8.

The third and last gene of the *nanCMS* operon, *nanS*, was previously thought to be a conditionally essential gene of *E. coli* for growth on glycerol as sole carbon source [88]. However, Steenbergen et al. [89] published evidence that NanS is a sialate O-acetyl esterase, indicating that the glycerol-growth defect previously reported [88] was almost certainly caused by an uncharacterized secondary mutation in the test strain. In other words, growth of newly constructed *nanS* mutants on Neu5,9Ac_2_ was eliminated while the mutant grew normally with glycerol [89]. Discerning the true function of NanS was made possible by two key observations: a commercially available source of Neu5,9Ac_2_ and a bioinformatics survey of *nanS* against the microbial genomic database which identified weak similarity to an acetyl xylan esterase (*axe*) [8]. Because esterases frequently share conserved primary structural similarities including active site residues [90], it was logical that NanS might be a sialate O-acetyl esterase because it mapped within a NanR-coregulated operon and was at least partly similar to Axe [88]. Remarkably, when NanS is screened against its close bacterial relatives none has a discernable copy of *nanS* despite the presence of genetic information known to or to potentially encode and regulate the canonical Neu5Ac dissimilatory pathway (Figures 8 and 9). Some of the species shown in Figure 9 that are related to *E. coli* include orthologs of *nanC*, *namM*, or *yjhBC* though none has a copy of *nanS* regardless of whether the database is screened against *nanS* or *axe* other than *Shigella dysenteriae* (see below). By contrast to the absence of nanS or axe orthologs in enteric bacteria closely related to E. coli, potential axe orthologs abound in GIT bacterial species (Tables 1 and 2), suggesting that an ability to metabolize O-acetylated sialic acids is a common phenotype of bacteria living on or at a mucosal surface [89]. One drawback working with commercially available O-acetylated sialic acids is their relative lack of purity such that preparations of Neu5,9Ac_2_ or Neu4,5Ac_2_ contain impurities including Neu5,(7,8)Ac_2_ contaminants [89]. Clarke et al. [91] recently reported the chemical synthesis of Neu2,5Ac_2_, Neu4,5Ac_2_, and Neu5(7,8)Ac_2_ derivatives in pure form. Relatively straightforward chemical synthetic methods for preparing O-acetylated sialic acids should facilitate future research on these interesting and phylogenetically widespread Neu5Ac derivatives. The identity of NanS as an O-acetyl esterase was recently confirmed by Rangarajan et al. [92], who presented a crystal structure of the NanS homolog from *E. coli* O157:H7. While there is nothing remarkable about the structure partial characterization of the NanS active site residues suggested NanS is the founding member of a subfamily of esterase [92].

Unlike *E. coli* with its three coregulated *nan* operons all known close relatives containing predicted NanR orthologs include only one or in the case of *P. haloplanktis* two *nan* operators (Figure 9). These observations predict a general lack of coordinated *nan* expression in species related to *E. coli*, and that only *E. coli* is capable of metabolizing O-acetylated sialic acids within this related bacterial group. Evidence that the latter conclusion is true came from an analysis of wild type *S. enterica *var Typhimurium (*S. typhimurium*) grown on Neu5Ac or Neu5,9Ac_2_, while *S. typhimurium* wild type grew as expected with Neu5Ac as sole carbon source, a result supported by the inability of a *nanA* mutant to grow under the same condition, the wild type did not grow when the O-acetylated sialic acid was provided as sole carbon source [89]. This last result is consistent with the predicted absence of *nanS* in *S. typhimurium* [89]. The clear implication of these results is that with the possible exception of *S. dysenteriae*, the *nan* regions of species shown in Figure 9 lack the genetic information to encode esterase or the ability to metabolize O-acetylated sialic acids. *S. dysenteriae*, the causative agent of dysentery, includes a gene with an internal domain paralogous to *nanS* and two predicted domains of unknown function at the N- or C-termini flanking the *nanS* paralog (Figure 9). Interestingly, the *nanS* paralog is located in the *S. dysenteriae* prophage that encodes shiga toxin. Indeed, the prophage copy of *nanS* immediately follows in the same transcriptional direction as the two genes encoding subunits of the holotoxin. One possibility for the close association of *nanS* with toxin genes is that the epithelial toxin receptor somehow involves the need to convert O-acetylated sialic acid(s) to Neu5Ac. Other prophage copies of *nanS* exist in E. coli O157 strains and other serotypes causing hemorrhagic disease (Table 3).

The prophage carrying shiga-like toxin in most EHEC strains is similar to the *S. dysenteriae* phage as are the encoded toxin (Stx or Stx-like) subunits. As shown in Table 3, some *stx*-positive bacteria are predicted to express a variable number of *nanS* paralogs, where short refers just to the *E. coli* K-12 homolog (Figure 8), long to *nanS* with N- and C-terminal domains, and partial to *nanS* plus one or the other flanking domain. Other strains of pathogenic *E. coli* from EAEC, ExPEC, and EPEC groups lack *stx* but may have multiple copies of *nanS* that are invariably associated with prophage remnants. Remarkably, one EHEC appears to lack even the NanR-regulated copy of *nanS* whereas 24 other sequenced strains, like *E. coli* K-12, lack *stx,* and *nanS* paralogs (Table 3) and 3 strains, ATCC8739, SE11, and UMNK88 lack any versions of *nanS*. This bioinformatics survey beggars many questions warranting future investigation. Do *nanS* paralogs have O-acetyl esterase activity? If so, why are seemingly redundant copies of *nanS* located in prophage or prophage remnants? Is expression of *nanS* essential for dysentery or hemorrhagic diseases; if so, why do some strains lack even the otherwise common *nanS* copy? Indeed, one strain lacks even the canonical NanR-regulated *nanATEK*-*yhcH* operon (Table 3). In other words, so many *E. coli* strains have already been or are being sequenced that it is possible to find nearly every conceivable variant of *nan* organization. Does this variation mean that some or all *nan* genes are nonessential to the *E. coli* lifestyle, or more likely that variants might be on their way to extinction or have partially different lifestyle than the majority of *E. coli* strains? What are the functions if any of the N- and C-terminal domains flanking *nanS* paralogs? Why is *nanS* absent in some bacteria with otherwise intact *nan* systems? Finally, is *E. coli nanS* really essential for the evolutionary success of this bacterium as a human and animal commensal, facultative, and sometimes frank pathogen? Determining the answers to some of these questions will surely increase understanding of sialometabolism and have the potential to suggest new ways of manipulating mucosal bacterial physiology in general.

As shown in Tables 1 and 2 and Figure 6, many bacterial GIT-inhabitants with predicted sialocatabolic systems include a copy of *nanS* (*axe*). This finding is consistent with a potentially important role of NanS in supporting the commensal lifestyle involving utilization of host-derived sialic acids other than Neu5Ac. For example, it is unclear why pneumococcal strains have distinct *nan* genetic organizations whereas all strains examined, like GBS, include one copy of *nanA* in their genomes (Figure 6). Unpublished data from the author's laboratory has shown that the *nanS* homologs in streptococci encode functional Neu5,9Ac_2_ O-acetyl esterases. The obvious experimental approach to extend these findings is to eliminate streptococcal esterase(s) and determine the effects on host colonization or disease. However, because the role of sialocatabolism in pneumococcal infection is suspect, the best candidate organism for the proposed studies is GBS, which seem to have a clearer dependency on sialocatabolism for colonization than pneumococci [45].

Compared to the at least partially characterized functions of NanATEK and NanCMS, little is known about *yhcH* or *yjhBC* except that these genes are coregulated by NanR in *E. coli* K-12 (Figure 8). Species closely related to *E. coli* have one or in the case of *E. tarda*, two *yhcH* copies, whereas *yjhB* and *yjhC* are infrequently detected. The YhcH ortholog in *H. influenzae* was purified and its crystal structure solved [93]. The resulting conjecture that it might function in catabolism of Neu5Gc was not supported when an *E. coli yhcH* null mutant was shown to grow as well as wild type on Neu5Gc [31]. However, solving the crystal structure of YhcH does support a possible epimerase activity [93]. Despite the absence of positive data, the similarity of YhcH to an epimerase, YjhB primary structure being similar to NanT, and YjhC primary structure suggesting it is a possible oxidoreductase (Figure 8), strongly suggests that like NanS, genes coregulated as part of the sialoregulon function in metabolism of sialates other than Neu5Ac or O-acetylated sialates. Note that *S. pealeana* and *P. haloplanktis* lack *yhcH* and *yjhB* (Figure 9), suggesting that the spectrum of sialates metabolized by these bacteria might be less than for most *E. coli* strains. Were a panel of all likely sialic acids present at mucosal surfaces available, it would be straightforward to determine all those derivatives of Neu5Ac metabolized by *E.coli* but not by *S. pealeana * or *P. haloplanktis.* Indeed, since some *E. coli* lack certain genes of the sialoregulon (Table 3), these strains alone might suffice to determine the functions of *yhcH* and *yjhBC*. Therefore, instead of waiting for chemical methods that would probably be available only to a few laboratories, simply isolating all sialates from selected mucosae and exposing them to *E. coli* and relevant mutants or natural mutant phenocopies could facilitate identification of all currently unknown gene functions, as long as the results are combined with simple chemical detection methods [5, 76].

Bacteria have evolved diverse sialate transport systems including symporters, ABC- and TRAP-transporters [3, 94]. NanT is a proton symporter with 14 instead of the usual 12 membrane spanning domains [3, 94]. By contrast, YjhB though similar to NanT lacks the central hydrophilic domain found in NanT [95]. This domain is thought to be essential for uptake of Neu5Ac, Neu5Gc, and certain other sialates [3, 26, 89]. Therefore, the presumed sialate(s) transported by YjhB should be structurally distinct from more common sialates and might have specificity for less common forms like ADOA or Neu5Ac1,7L (Figures 3(b) and 3(c), resp.). As shown in Figure 4, Neu5Ac1,7L seems to be a relatively common sialate in the large intestine, suggesting it could be a potentially important source of bacterial nutrition. Chemical synthesis of sialyl lactones has been reported [96], suggesting simple experiments to determine its utilization by *E. coli* and possibly identify the function of *yjhB*. ADOA is an oxidized form of Neu5Ac that may serve as an essential hydroxyl free radical scavenger in tissues [97, 98]. 

**Figure 10:**
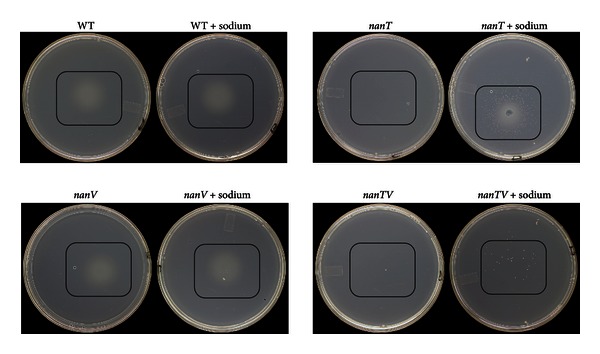
Auxanographic analysis of Neu5Ac utilization by *S. typhimurium nanT* and *nanV* mutants. The indicated strains were grown in minimal medium with glycerol as sole carbon source and plated in top agar with no carbon source and with or without 100 mM sodium chloride. Black rectangles indicate areas where Neu5Ac was added, with growth shown by the hazy zones or individual colonies.

*S. typhimurium* is closely related to *E. coli* but has only one predicted operon regulated by NanR (Figure 9). However, immediately downstream of a duplicated copy of *nanE* (ManNAc-6-P to GlcNAc-6-P epimerase) is a predicted sodium-solute symporter that was shown to complement an *E. coli nanT* mutant for growth on Neu5Ac in trans [99]. This result suggests that *S. typhimurium *spends at least some of its time in an environment with at least physiological levels (*c.* 140-mM) of sodium, concentrations found commonly in all human or other animal hosts. The problem with the complementation study is no evidence was presented showing the sodium-solute sialate symporter (here designated *nanV*) in fact functions as such in *S. typhimurium *[99].  Figure 10 shows the results of an auxanographic analysis of *S. typhimurium* strain 14028 wild type, *nanT*, *nanV* and *nanTV* double mutant growth on Neu5Ac as sole carbon source. Auxanography is a common procedure where bacteria suspended in soft (0.7%) agar are plated on top of 1.5% bottom agar, both lacking at least one essential growth factor [100, 101]. The analysis can be carried out qualitatively by sprinkling about 1 mg of substrate at one point of the plate, or semiquantitatively by applying a precise amount either in a small liquid volume or onto a paper disk [26]. The results as expected show growth of the wild type (WT) on Neu5Ac and none by the *nanT* mutant. However, whereas growth was observed for the WT on a plate where the top agar was supplemented with 100 mM sodium, similar growth was observed for the *nanT* mutant demonstrating the sodium-dependency of another sialate uptake system. The sodium-dependent phenotype of the *nanT* mutant was lost when a *nanTV* double mutant was plated in the presence or absence of sodium (Figure 10). However, some few colonies observed in the double mutant with sodium suggest another sodium-sialate transporter remains to be identified. It will be interesting to test this isogenic mutant series for fitness defects in animal models of salmonellosis. These studies are in progress.

## 9. How Bacteria Catabolize Neu5Gc and Methylated Sialic Acid (Neu5AcMe)

**Figure 11:**
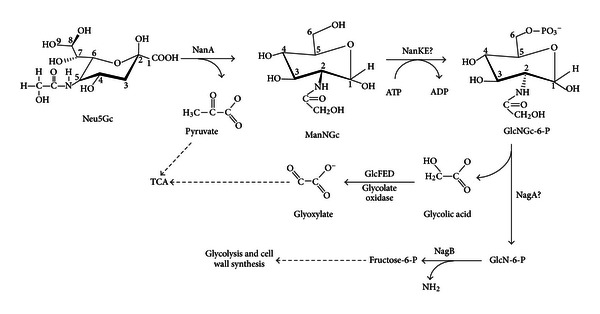
Proposed pathway for utilization of Neu5Gc by *E. coli*. After transport by NanT, Neu5Gc is degraded to pyruvate and *N*-glycolylmannosamine (ManNGc) by NanA. It is proposed that the combined actions of NanK and NanE convert ManNGc to *N*-glycolylglucosamine-6-phosphate (GclNGc-6-P). The rest of the pathway combines the actions of NagAB with the glyoxylate shunt to complete catabolism of Neu5Gc. This pathway implies that the hydroxyl group of Neu5Gc does not impede already known enzymatic activities as described in the text.

It has been known since 1985 that *E. coli* uses Neu5Gc as a sole carbon source [26]. Although as discussed above this sialic acid is not synthesized by humans it is found in most other animal hosts where it could serve an important nutritional source for bacterial colonization. Because Neu5Gc differs from Neu5Ac by a single hydroxyl group at the carbon-5 position of Neu5Ac (Figure 3(a)), there might be an enzyme that first removes the group before or after transport of Neu5Gc by NanT. However, no such enzyme is known to exist in nature indicating that Neu5Gc metabolism probably begins with cleavage by NanA to release pyruvate and ManNGc. This activity of the sialate lyase has been demonstrated biochemically for the mammalian homolog of NanA [102]. Since we know Neu5Gc serves as a sole carbon source for *E. coli* and is cleaved by NanA, how *E. coli* handles the resulting hydroxylated ManNAc derivative, ManNGc, should define the pathway for catabolism of Neu5Gc in *E. coli* and probably all other bacteria that catabolize this substrate. In a preliminary experiment from the author's laboratory the expected accumulation of Neu5Gc by an *E. coli nanA* mutant was confirmed by previously described chemical methods [76], demonstrating that the hydroxyl, as expected, is stable after uptake, that is, there is no dehydroxylase in the cell.  Figure 11 shows that once Neu5G is cleaved by NanA, the resulting ManNGc is likely to be phosphorylated by NanK and epimerized by NanE to yield *N*-glycolylglucosamine-6-phosphate (GlcNGc-6-P). If the NagA deacetylase can remove the glycolyl group, yielding GlcN-6-P, then NagB would complete the pathway by converting GlcN-6-P to fructose-6-P just as it does in the canonical Neu5Ac pathway (Figure 5). The remaining glycolic acid would then be a substrate for the *glc* system, which is induced by glycolate, yielding glyoxylate [103]. Glyoxylate can then either be condensed with acetyl coenzyme-A by malate synthase G, or two molecules acted upon by glyoxylate carboligase, which simultaneously decarboxylates the condensation product, tartronic semialdehyde, and reduces it to glycerate that is then phosphorylated to glycerate-3-phosphate. These reactions constitute what is known as the glycerate pathway [103]. The *E. coli* pathway proposed for metabolism of Neu5Gc in Figure 11 is straightforward to verify as it involves readily available methods of bacterial mutagenesis of known target genes. If Neu5Gc is an important carbon source in vivo, a mutant defective in glycolate oxidase might have an interesting phenotype that should be easy to determine. A variant of the pathway proposed in Figure 11 has been speculated upon in mammalian cellular metabolism of Neu5Gc [104]. 

Neu5AcMe is sialic acid with a methyl group attached to the carbon-1 carboxylate group (Figure 3(a)). *E. coli* uses this sugar as sole carbon source despite the inability of NanA to cleave methylated sialic acid [26]. On the basis of pervious studies with NanS [89], it is reasonable to conclude that an as yet unidentified methyl esterase(s) exists in the periplasm of *E. coli* to convert the methylated sialate to Neu5Ac. A pattern of metabolism similar to that of *N*-glycolyl or methyl group removal is envisioned for other sialates with lactyl or other simple chemical substitutions found in the GIT (Figure 4). The picture emerging from the admittedly still limited number of studies concerning the sialoregulon is that bacteria and especially *E. coli *have evolved metabolic functions to funnel the diversity of host-derived sialates to Neu5Ac or other readily digestible forms of this sugar. This dataset suggests a simple model of sialocatabolism for at least some of the sialoregulon parts that should be universally true for all bacteria with homologous sialocatabolic functions. 

## 10. Model of Known or Proposed Sialocatabolic Pathways in *E. coli*

**Figure 12:**
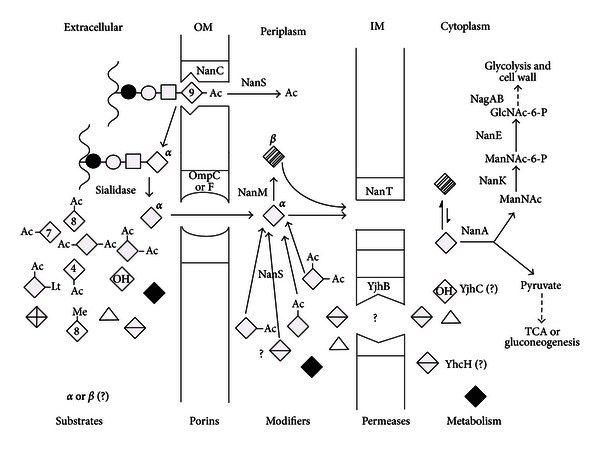
Known or proposed functions of the *E. coli* sialoregulon. Wavy lines indicate mucin peptide backbones with idealized O-linked (serine/threonine) oligosaccharides composed of GalNAc (bold circle); GlcNAc (open squares); Gal (open circles); Neu5Ac (open diamonds, indicating the alpha anomer while hatched diamonds represent the thermodynamically favored beta anomer). Neu5Ac derivatives: acetyl (Ac) groups indicated by their linkages to different Neu5Ac carbon position (numbers in diamonds); Neu5,9Ac_2_, Neu5,7Ac_2_, Neu5,8Ac_2_, Neu4,5Ac_2_, Neu5,7,9Ac_3_, Neu5,8,9Ac_3_, and Neu5Gc (diamond with OH) is Neu5Ac with hydroxyl at position 5 (see Figure 1), Lt (lactyl), Me (methy). Bold diamond is neuraminic acid (Neu, which lacks the carbon-5 acetamido group). Triangle represents ADOA while diamond with horizontal line represents Neu5Ac1,7L (see Figures 3(b) and 3(c)). Diamond with cross lines represents Neu5Ac2en (see Figure 3(d)). Other abbreviations are as given in the legend to Figure 7.

The model is subdivided into five parts specifying the various *E. coli* cellular compartments: extracellular space, outer membrane (OM), periplasm or periplasmic space, inner membrane (IM), and cytoplasm (Figure 12). The various components of the sialocatabolon are then either substrates, porins allowing ingress of substrates to the periplasm where modifiers convert sialate derivatives into forms transportable by NanT or YjhB (permeases) located in the IM, and further conversion cytoplasmically as needed to Neu5Ac, followed by the actions of the canonical metabolic pathway (Figure 5). 

Sialates released from sialoglycoconjugates by endogenous or exogenous sialidase(s) are immediately available for passive diffusion into the periplasm through outer membrane pores (porins) OmpC, OmpF, and NanC with molecular weight exclusion sizes of about a disaccharide. Though NanC is unessential for growth on Neu5Ac it might allow certain oligosaccharides with O-acetylated sialic acids to gain access to NanS in the periplasm, which would release the acetyl group(s) thus facilitating conversion in the extracellular milieu to free sialic acids. This idea is easy to test because NanS modifies glycoketosidically linked O-acetylated sialic acids as well as the free sugars. Therefore, a strain with a fully induced sialoregulon when exposed to SGM would allow one to determine if NanC facilitates oligosaccharide deacetylation. Alternatively, NanC might simply facilitate ingress of one or more of the minor sialates not recognized well by the major porins. Once in the periplasm NanM, NanS, and other unidentified modifying enzymes facilitate conversion of sialates to form(s) recognized by NanT and, possibly, YjhB. After transport to the cytoplasm other reactions presumably occur that convert any remaining sialates to Neu5Ac for subsequent cleavage to pyruvate and ManNAc by NanA. The resulting ManNAc, pyruvate, and glycolate in the case of Neu5Gc are then available for final conversion to carbon, nitrogen, and energy sources or cell wall precursors. Sialocatabolism thus is capable of fulfilling all cellular metabolic needs, consistent with its widespread occurrence and diversity in mucosal bacteria.

## 11. Conclusions

Any new or original idea goes through at least three stages: first many say it is not true, then they say it is true but not interesting; finally, it is deemed true and interesting but not new (paraphrased from “anonymous”). It is only when a new scientific field reaches the third ideation that it has any chance of attracting adherents and the subsequent funding necessary for expansion. The field of microbial sialobiology began in 1985 with discovery of a sialic acid catabolic system in *E. coli *[26, 27]. At that time most microbiologists had no idea what sialic acids were, and when explained to them they said they are “just another sugar” of no particular interest or importance. Those few researchers who knew something about sialic acid structural and phylogenetic diversity either thought the sugars were either too unstable or otherwise inaccessible to have any special relevance to microbial growth and overall bacterial physiology, and so the field has had a long gestation. The current paper focused on advances in microbial sialobiology since the field was last reviewed in 2004 [3]. Major advances since then have been the expanding knowledge of the sialoregulon and tantalizing in vivo experiments supporting minor to definitive roles of sialometabolism in diverse host-microbe interactions. These recent findings are quite separate from the well-known functions of host sialic acids as microbial or toxin adhesins or regulators of innate immunity, knowledge of which has had little success generating practical advances in biomedicine. By contrast, targeting *E. coli* K1, certain neisserial serotypes, *Haemophilus* spp, and *P. multocida* synthetic or hybrid catabolic systems of sialic acid surface decoration are already known to have therapeutic potential [3, 5, 34, 35, 48, 51, 105]. On the basis of these practical advances and the basic theoretical and experimental underpinnings the pace of research in microbial sialobiology is likely to increase. Therefore, the point of the current paper is to increase optimism by presenting a coherent unified theory of bacterial sialometabolism. Certainly some ideas will not withstand detailed scrutiny. Indeed, for reasons described some of the in vivo studies are not even likely to be reproducible. All the likely failures should stimulate rather than impede attempts to sharpen experimental approaches. It is hoped that the basic scientific findings presented in this paper will stimulate the proper scrutiny and help guide the field during its mature stage.

## Figures and Tables

**Table 1:** Some of the bacterial species isolated from the healthy human intestine.

Microorganism (recent designation)	*E* value^a^	Maximum percent identity^b^
Gram-negative anaerobic rods		
* Bacteroides asaccharolyticuslyitalics (Porphyromonas asaccharolyticus) *	0.063	43% (46/106)
* Bacteroides capillosus *	4 × 10^−6^	24% (43/180)
* Bacteroides distasonis (Parabacteroides distasonis) *	3 × 10^−5^	25% (50/203)
* Bacteroides eggerthii *	3 × 10^−5^	23% (70/311)
* Bacteroides fragilis *	8 × 10^−6^	26% (58/222)
* Bacteroides furcosus *	No similarity found	
* Bacteroides hypermegas (Megamonas hypermegale) *	0.023	32% (23/93)
* Bacteroides melaninogenicus* subsp. *mel. (Prevotella melaninogenica) *	0.002	25% (30/120)
* Bacteroides multiacidus (Mitsuokella multacida) *	0.004	35% (16/46)
* Bacteroides ovalis *	No sequence available	
* Bacteroides ovatus *	2 × 10^−29^	28% (83/294)
* Bacteroides praeacutus (Tissierella praeacuta) *	0.88	50% (7/14)
* Bacteroides putredinis (Alistipes putredinis) *	0.002	31% (36/117)
* Bacteroides ruminicola* subsp.* brevis *	No similarity found	
* Bacteroides ruminicola* subsp.* ruminicola (Prevotella ruminicola) *	1 × 10^−27^	28% (80/287)
* Bacteroides splanchinicus (Odoribacter splanchnicus) *	5 × 10^−4^	25% (33/131)
* Bacteroides thetaiotaomicron *	6 × 10^−30^	28% (84/305)
* Bacteroides uniformis *	2 × 10^−23^	26% (80/314)
* Bacteroides vulgatus *	1 × 10^−28^	28% (82/297)
* Desulfomonas pigra (Desulfovibrio piger) *	0.001	31% (15/48)
* Leptotrichia buccalis *	0.002	27% (30/111)
* Fusobacterium mortiferum *	0.001	27% (33/124)
* Fusobacterium naviforme *	No similarity found	
* Fusobacterium necrogenes *	No similarity found	
* Fusobacterium nucleatum *	0.014	44% (11/25)
* Fusobacterium plauti (F. plautii) *	0.003	39% (17/44)
* Fusobacterium prausnitzii (Faecalibacterium prausnitzii) *	8 × 10^−5^	22% (33/153)
* Fusobacterium russi (F. russii) *	None found	
* Fusobacterium symbiosum *	No sequence available	
* Fusobacterium varium *	8 × 10^−79^	40% (124/310)
* Butyrivibrio fibriosolvens *	4 × 10^−5^	22% (29/131)
* Sucinimonas amylolytia *	No sequence available	
* Vibrio succinogenes *	0.055	31% (25/81)
Gram-positive anaerobic rods		
* Bifidobacterium adolescentis *	0.018	26% (37/143)
* Bifidobacterium angulatum *	9 × 10^−4^	56% (14/25)
* Bifidobacterium bifidum *	0.005	22% (25/114)
* Bifidobacterium breve *	0.012	50% (13/26)
* Bifidobacterium catenulatum *	0.084	43% (9/21)
* Bifidobacterium cornutum *	No sequence available	
* Bifidobacterium dentium *	0.016	29% (24/82)
* Bifidobacterium infantis (B. longum *subsp.* infantis) *	0.005	52% (13/25)
* Bifidobacterium longum *	0.006	52% (13/25)
* Bifidobacterium pseudolongum (B. longum* subsp. *longum) *	0.006	52% (13/25)
* Clostridium bejerinkii (C. bejirinckii) *	3 × 10^−5^	20% (41/209)
* Clostridium butyricum *	1 × 10^−76^	31% (123/402)
* Clostridium cadaveris *	3.3	50% (4/8)
* Clostridium celatum *	No similarity found	
* Clostridium clostridiiforme *	0.047	21% (17/80)
* Clostridium difficile *	0.023	28% (41/148)
* Clostridium innocum *	0.72	22% (11/50)
* Clostridium leptum *	0.026	25% (18/76)
* Clostridium malenominatum *	2.9	67% (4/6)
* Clostridium nexile *	1 × 10^−4^	43% (20/47)
* Clostridium paroputrificum *	1.2	20% (15/77)
* Clostridium perfringens *	4 × 10^−83^	43% (132/310)
* Clostridium ramosum *	0.11	28% (17/60)
* Clostridium tertium *	0.13	26% (19/72)
* Eubacterium aerofaciens (Collinsella aerofaciens) *	1 × 10^−54^	34% (108/314)
* Eubacterium contortum *	No similarity found	
* Eubacterium cylindroides *	0.042	32% (13/41)
* Eubacterium lentum (Eggerthella lentum) *	0.064	32% (22/68)
* Eubacterium limosum *	0.003	32% (23/74)
* Eubacterium rectale *	0.018	26% (14/53)
* Eubacterium ruminantium *	2.5	36% (5/14)
* Eubacterium tenue *	No sequence available	
* Eubacterium tortuosum *	No similarity found	
* Eubacterium ventriosum *	1 × 10^−4^	41% (22/54)
* Lachnospira multiparus *	1.6	60% (6/10)
* Propionibacterium acnes *	1 × 10^−20^	25% (78/313)
* Propionibacteriu granulosum *	4.1	71% (5/7)
* Propionibacterium jensenii *	0.25	30% (17/57)
Anaerobic cocci		
* Acidaminococcus fermantans (A. fermentans) *	0.16	31% (18/58)
* Megasphera elsdenii (Megasphaera elsdenii) *	0.14	34% (11/32)
* Peptococcus asaccharolyticus (Peptoniphilus asaccharolyticus) *	0.023	49% (19/39)
* Peptococcus magnus (Finegoldia magna) *	6 × 10^−4^	30% (17/57)
* Peptococcus prevotii (Anaerococcus prevotii) *	3 × 10^−51^	34% (98/291)
* Peptostreptococcus productus *	No similarity found	
* Ruminococcus albus *	4 × 10^−8^	28% (41/149)
* Ruminococcus bromii *	0.046	37% (14/38)
* Ruminococcus flavefaciens *	4 × 10^−6^	26% (39/152)
* Sarcina ventriculi *	0.76	32% (8/25)
* Streptococcus constellatus *	0.039	28% (14/50)
* Streptococcus intermedius *	5 × 10^−6^	23% (42/182)
* Streptococcus morbillorium (Gemella morbillorum) *	6 × 10^−61^	35% (105/247)
* Veillonella parvula *	0.014	23% (28/120)
* Coprococcus cutactus *	No sequence available	
* Coprococcus catus *	0.022	29% (20/68)
* Coprococcus comes *	0.004	26% (16/61)
* Gemiger formicilis *	No sequence available	
Facultatively anaerobic bacteria		
* Enteric bacteria *		
* Escherichia coli *	9 × 10^−6^	26% (35/137)
* Citrobacter freundii *	2 × 10^−4^	23% (57/251)
* Klebsiella pneumonia *	8 × 10^−5^	24% (34/140)
* Enterobacter cloacae *	6 × 10^−4^	23% (30/133)
* Enterobacter aerogenes *	0.006	24% (60/249)
* Proteus mirabilis *	3 × 10^−4^	42% (20/48)
* Proteus morganii *	0.15	41% (15/37)
Lactobacilli		
* Lactobacillus acidophilus *	6 × 10^−4^	25% (36/144)
* Lactobacillus brevis *	0.012	26% (14/55)
* Lactobacillus casei *	0.038	26% (28/107)
* Lactobacillus fermentum *	0.011	22% (11/49)
* Lactobacillus leichmannii (L. leichmanni) *	3.9	29% (8/28)
* Lactobacillus minutus *	2.8	67% (6/9)
* Lactobacillus plantarum *	0.089	24% (29/121)
* Lactobacillus rogosae *	No similarity found	
* Lactobacillus ruminis *	4 × 10^−13^	37% (29/78)
* Lactobacillus salivarius *	0.062	34% (16/47)
Streptococci		
* Streptococcus faecalis (Enterococcus faecalis) *	1 × 10^−81^	43% (128/301)
* Streptococcus faecium (Enterococccus faecium) *	2 × 10^−81^	43% (128/301)
* Streptococcus bovis *	2 × 10^−99^	48% (152/319)
* Streptococcus agalactiae *	1 × 10^−124^	55% (172/313)
* Streptococcus anginosus *	9 × 10^−4^	43% (20/47)
* Streptococcus avium (Enterococcus avium) *	0.82	28% (10/36)
* Streptococcus cremoris (Lactococcus lactis* subsp. *cremoris) *	1 × 10^−77^	40% (124/311)
* Streptococcus equisimilius (S. dysgalactiae* subsp. *equisimilis) *	0.004	29% (19/65)
* Streptococcus lactis *	2 × 10^−78^	41% (125/307)
* Streptococcus mitior (S. mitis) *	0.0	94% (307/326)
* Streptococcus mutans *	0.001	26% (38/148)
* Streptococcus salivarius *	0.001	31% (37/121)
* Streptococcus sanguis (S. sanguinis) *	0.0	93% (303/326)
* Staphylococcus epidermidis *	0.111	22% (16/73)

^a^Sequenced microbial genomes of some of the species found in the healthy human intestine with recent designations given in parentheses [6]. Genera were queried for similarity to acetyl xylan esterase [8]. The lower the expected value (*E*) is reflects the likelihood that a match is not due to chance.

^b^Indicates the percentage of identical amino acids within the specified alignment length. The number of identical amino acids/the alignment length is given in parentheses.

**Table 2:** Additional bacterial species isolated from the healthy intestines of nonhuman animals.

Microorganism (recent designation)^a^	*E* value	Maximum percent identity
Gram-negative anaerobic rods	0.018	27% (12/44)
*Bacteroides succinogenes *		
(*Fibrobacter succinogenes subsp. succinogenes*)*		
*Bacteroides ruminicola (Prevotella ruminicola*)*	1 × 10^−27^	28% (80/287)
*Bacteroides amlylophilus (Ruminobacter amylophilus*)*	1.3	25% (13/51)
* Bacteroides termitidis (Sebaldella termitidis) *	0.013	34% (21/61)
* Bacteroides clostridiiformis (Clostridium clostridioforme) *	0.047	21% (17/80)
Succinivibrio dextrinosolvens *	1	50% (5/10)
*Selenomonas ruminatium (S. ruminantium* subsp. lactilytica) *	5 × 10^−4^	32% (19/59)
*Desulfovibrio* spp.	3 × 10^−4^	35% (20/57)
*Desulfotomaculum nigrificans *	0.004	30% (13/44)
Desulfotomaculum ruminis *	0.004	30% (18/61)
*Desulfotomaculum orientis *	No similarity found	
*Oscillospira guillermondii *	No similarity found	
Gram-positive anaerobic rods		
Eubacterium cellulosolvens *	7 × 10^−4^	50% (19/38)
*Eubacterium nitrogenes (Eubacterium nitritogenes) *	No similarity found	
* Eubacterium helminthoides *	No sequence available	
* Cellulomonas flavigena *	7 × 10^−35^	29% (97/330)
* Brevibacterium *spp.	no similarity found	
* Bifidobacterium asteroides *	0.66	67% (8/12)
* Bifidobacterium indicum *	0.41	35% (9/26)
* Bifidobacterium coryneforme *	0.54	67% (8/12)
Bifidobacterium pseudolongum*	0.63	34% (11/32)
Bifidobacterium ruminale (Bifidobacterium thermophilum)*	1.3	75% (6/8)
Lactobacillus ruminis*	4 × 10^−13^	37% (29/78)
Lactobacillus vitulinus*	0.6	42% (5/12)
Clostridium lochheadii*	No sequence available	
Clostridium longisporum*	0.97	43% (9/21)
Clostridium cellobioparum*	2.2	64% (7/11)
* Methanobacterium formicicum *	0.53	55% (6/11)
*Methanobacterium ruminantium (Methanobrevibacter ruminantium) *	0.089	36% (10/28)
*Methanobacterium mobile *	No similarity found	
Anaerobic cocci		
*Methanosarcina barkeri *	1 × 10^−7^	22% (40/181)
Lampropedia hyalina *	No similarity found	
*Veillonella alcalescens *	0.014	23% (28/120)
Facultatively anaerobic bacteria		
*Salmonella* spp.	4 × 10^−5^	26% (35/137)
*Hafnia alvei *	0.016	27% (14/52)
*Streptococcus equinus (Streptococcus bovis) *	2 × 10^−99^	48% (152/319)
*Lactobacillus lactis (Lactobacillus delbrueckii* subsp. *lactis) *	0.001	25% (42/165)
*Lactobacillus buchneri *	0.045	27% (20/73)
*Lactobacillus cellobiosus (Lactobacillus fermentum) *	0.011	22% (11/49)
Miscellaneous bacteria		
*Borrelia* spp.	0.017	45% (9/20)
* Bacillus macerans (Paenibacillus macerans) *	0.055	31% (12/39)
* Acholeplasma bactoclasticum *	No similarity found	
* Gemmiger formicilis *	No similarity found	
* Alcaligenes faecalis *	0.003	28% (13/47)
*Fusosporus* spp.	No similarity found	
*Arthromitus* spp. *(Candidatus arthromitus) *	6 × 10^−4^	27% (24/88)
* Anisomitus* spp.	No sequence available	
* Entomitus* spp.	No sequence available	
* Coleomitus* spp.	No sequence available	
* Bacillospira* spp.	No sequence available	
* Sporospirrillum* spp.	No sequence available	
* Metabacterium* spp.	3 × 10^−8^	25% (40/159)

^a^Bacteria isolated from nonhuman animal intestines with newer designations given in parentheses. Asterisks indicate bacteria isolated mainly or exclusively from the rumen [6]. Expect values (*E*) and maximum percentage identities are as described in Table 1.

**Table 3:** Distribution of *nanS* and *stx* in sequenced *E. coli * strain.

Strain	Copies of *nanS *	Copies of *stx *
Enterohemorrhagic *E.coli *(EHEC)		
O23:H11 11368	1 short, 10 long, 1 broken into 2 pieces of 108 aa and 513 aa	1
O26:H11	1 short, 11 long	1
O103:H2 12009	1 partial, 1 short, 1 med., 7 long	2
O104:H4	1 med., 4 long	2
O111:H^-^ 11128	1 short, 10 long	2
O145:H28	1 long	1
O157:H7 EDL933	1 short, 5 partials, 7 long	2
Enteroaggregative *E.coli *(EAEC)		
55989	3 long	0
Extraintestinal pathogenic *E. coli *(ExPEC)		
CFT073 (K2)	2 short	0
NA114	1short, 1 very long	0
O7:K1 CE10	1 short, 2 long	0
UMN026 (K1)	2 short	0
Enteropathogenic *E. coli *(EPEC)		
E110019	1 short, 2 long	0
B171	1 short, 3 long	0
O55:H7 CB9615	1 short, 6 long	0
O127:H6 E2348/69	1 short, 3 long	0
Enterotoxigenic *E. coli* (ETEC)		
H10407	1 short	0
UMNF18	1 short	1
Other *E. coli *		
E22	1 short, 3 long	0
B088	2 short, 1 long	0
83972	2 short, 1 long	0
S88	1 short, 1 long	0
ED1a	2 short, 6 long	0
NA114	1 short, 1 long	0
101-1	1 short	0
536	1 short	0
AA86	1 short	0
ABU83972	1 short	0
APEC01	1 short	0
B185	1 short	0
B354	1 short	0
B7A	1 short	0
Bl21	1 short	0
BW2952	1 short	0
E24377A	1 short	0
F11	1 short	0
FVEC1412	1 short	0
HS	1 short	0
IAI1	1 short	0
IAI39 (O7:K1)	1 short	0
IHE3034	1 short	0
K-12 DH10B	1 short	0
K-12 MC1655	1 short	0
K-12 W3110	1 short	0
KO11FL	1 short	0
LF82	1 short	0
IO83:H1 NRG 857C	1 short	0
REL606	1 short	0
SE15	1 short	0
SMS-3-5	1 short	0
UM146	1 short	0
UT189	1 short	0
ATCC8739	0	0
SE11	0	0
UMNK88	0	0
